# The effect of Tai Chi lower extremity exercise on the balance control of older adults in assistant living communities

**DOI:** 10.1186/s12906-024-04382-9

**Published:** 2024-03-06

**Authors:** Min Mao, Vicki S. Mercer, Fuzhong Li, Michael T. Gross, Troy Blackburn, Bing Yu

**Affiliations:** 1 Division of Physical Therapy, The University of North Carolina at Chapel Hill, CB #7135 Bondurant Hall, Chapel Hill, NC 27599-7135 USA; 2 Shandong University, School of Hursing and Rehabilitation, Jinan, Shandong 250012 China; 3 Oregon Research Institute, 1715 Franklin Blvd., Eugene, OR 97403 USA; 4 Department of Exercise and Sport Science, University of North Carolina at Chapel Hill, Chapel Hill, NC 27599-8700 USA

**Keywords:** Postural balance, Physical functional performance, Aged, Tai Ji

## Abstract

**Background:**

Although Tai Chi (TC) is an evidence-based fall prevention training for older adults, its effective movements remain unclear, which may limit the practice of TC. The purpose of this study was to compare the effectiveness of TC lower extremity exercise (TC LEE), the 8-form Tai Chi (8-form TC), and a stretching control intervention for improving balance and functional mobility among older adults.

**Methods:**

This was a randomized controlled trial. A total of 102 participants (79 ± 6 years old) were recruited from assisted living facilities. All participants were randomly assigned to the TC LEE (*n* = 40), 8-form TC (*n* = 31), and stretching (*n* = 31) groups in which they received the respective interventions for 16 weeks. The Berg Balance Scale (BBS), Timed Up and Go (TUG) test, and center of pressure (COP) measurements during quiet stance were collected prior to and following the 16-week interventions. Comparisons on all measurements were conducted among all groups.

**Results:**

Significant improvements were found in BBS (*P* = 0.002), TUG test (*P* = 0.001), root mean square amplitude of COP displacement in the anterior–posterior (*P* = 0.001) and medial–lateral (*P* = 0.001) directions, and average COP speed in the anterior–posterior (*P* = 0.001) and medial–lateral (*P* = 0.001) directions after training in the TC intervention groups compared with the stretching group. The upper limit of the 95% confidence interval (CI) of differences in change scores on the BBS (-0.8 – 1.3 points) between the TC LEE group and the 8-form TC group was within equivalence margins (1.8 points), while the upper limit of the 95% CI of differences in change scores on the TUG test (0.1 – 2.1 s) exceeded the equivalence margin (0.7 s) with the TC LEE group having the larger change scores.

**Conclusion:**

TC LEE can improve balance and functional mobility in older adults, and may have greater effect than the 8-form TC on improving functional mobility as measured by the TUG test.

**Trial registration:**

ChiCTR2300070600 retrospectively registered.

**Supplementary Information:**

The online version contains supplementary material available at 10.1186/s12906-024-04382-9.

## Introduction

Falls are a serious threat to the health and well-being of older adults. About 30% of older adults (65 years of age or older) fall at least once a year [1]. The consequences of falls are disruptive to older adults’ independence and community participation. Among older adult fallers, 68% sustain fall-related physical injuries [2, 3]. Falls also result in a significant financial burden to individuals as well as to society. The medical costs for fall-related injuries in the United States in 2015 were over $40 billion [4]. Reducing the incidence of falls in older adults is important for improving their quality of life and reducing the financial burden to society.

Practicing Tai Chi is an effective way to prevent falls in older adults. This type of exercise consists of a series of slow movements performed in semi-squat positions with an emphasis on coordination of whole body movement and mind [5, 6]. Previous studies have demonstrated that practicing Tai Chi reduced risk of falls in older adults as indicated by decreased falls incidence [7–9] and by improvements in clinical measures such as the Berg Balance Scale (BBS) and the Timed Up and Go (TUG) test [10–12]. In addition, healthy community-dwelling older adults who practiced Tai Chi also had reduced center of pressure (COP) displacement and speed during bipedal and unipedal stance compared with older adults who exercised regularly but who did not practice Tai Chi [13–15].

Although practicing Tai Chi has positive effects on balance in older adults, most of the original Tai Chi exercises may be overly complicated to learn for older adults, especially for individuals with compromised cognitive capabilities. In clinical practice, older adults report that they spend too much effort remembering sequences of movements instead of focusing on balance and coordination when practicing Tai Chi. They may experience frequent pauses to allow recall of movements that connect one posture to the next [16]. Tai Chi has been used as a generic fall prevention program without considering that different Tai Chi movements may have different influences on the balance control of older adults, and that older adults with different capabilities may respond differently to Tai Chi movements. All above might compromise the effect of Tai Chi on improving balance in older adults [17, 18].

Efforts have been made to simplify Tai Chi exercises while maintaining the positive effects on fall prevention in older adults [19–21]. Li et al. simplified Yang Style Tai Chi by reducing the total number of forms to 8 from its original 24 forms (8-form Tai Chi) [16, 22]. This simplified Tai Chi exercise effectively reduced the number of falls/risk of falls and improved balance in healthy community-dwelling older adults [7, 22]. Body movements in Tai Chi exercise are characterized by: (1) maintaining a semi-squat position with the knees flexed during the entire exercise, (2) body movements with ankle dorsiflexion and plantarflexion, and (3) frequent body weight shifts from one lower extremity to the other [23]. These characteristics are closely related to the lower extremity movements of Tai Chi, which could potentially challenge the balance control of older adults, thereby improving the control of bodyweight over the base of support and increasing the proprioceptive function and muscle strength of the lower extremities. Practicing these lower extremity movements might improve the balance control of older adults, thereby, decreasing their risk of falls. However, no study has been done to confirm these ideas [22, 24, 25]. However, no study has been done to confirm these ideas.

In an effort to understand the effects of Tai Chi exercise on reducing the risk of falls for older adults and to enhance the clinical benefits of this exercise for fall prevention in older adults, we recently further simplified the 8-form Tai Chi (8-form TC) to a Tai Chi lower extremity exercise (TC LEE). The TC LEE involves only the lower extremity movements of the 8-form TC. Comparison between 8-form TC and TC LEM may provide insights concerning the characteristics of Tai Chi that are important for fall prevention. The purpose of this study was to compare the effectiveness of TC LEE, the 8-form TC, and a stretching control intervention for improving balance and functional mobility among older adults. We hypothesized that older adults who engaged in TC LEE training and 8-form TC training would display significant improvements in BBS and TUG test scores and in static postural control in quiet standing compared with the stretching group. We also hypothesized that improvements in the BBS and TUG tests would be similar in older adults who engaged in TC LEE training and 8-form TC training.

## Methods

### Participants

**Fig. 1:**
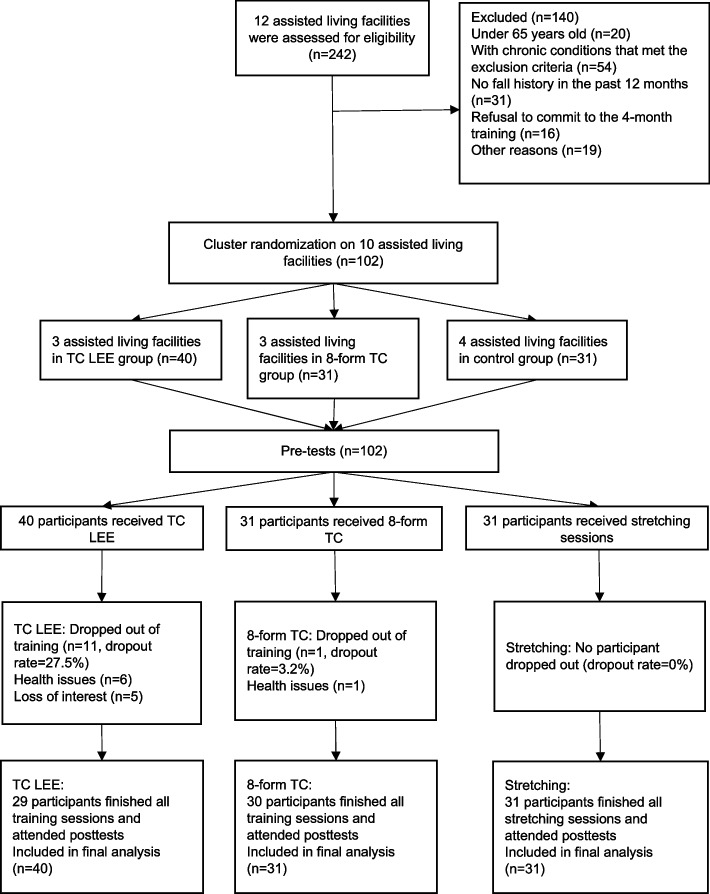
Recruitment and study design flow chart

A total of 242 individuals were contacted and screened, and 102 participants from local assisted living facilities were included in this study. The random assignment of participants to the 3 groups was detailed in Fig. 1. The 102 participants who participated in the study lived at 10 different assisted living facilities. All qualified participants at an individual facility were assigned to the same study group. The random assignment of facilities resulted in all participants at 3 facilities (*n* = 40) being assigned to the TC LEE group. All participants at 3 other facilities (*n* = 31) were assigned to the 8-form TC group. Finally, all participants at the remaining 4 facilities (*n* = 31) were assigned to the stretching group.

Inclusion criteria were that subjects: (1) were independently ambulatory without assistive devices; (2) were free of chronic medical problems, such as Parkinson disease, cardiovascular disease, stroke, and chronic pain that would limit participation in low-to-moderate intensity exercise; (3) had 2 or more falls in the past year; (4) had no cognitive impairment (Mini-Mental State Examination score ≥ 24) [26]; and (5) agreed to be assigned randomly to different groups and were willing to complete a 16-week training program. Exclusion criteria were that subjects: (1) had prior Tai Chi experience; (2) were involved in any moderate or strenuous activity more than 2 times a week on average less than 3 months prior this study; (3) had fracture within 6 months prior to the study; and a written consent was obtained from each participant before any data were collected. The use of human participants in this study was approved by the Institutional Review Board of the University of North Carolina at Chapel Hill, Chapel Hill, NC, USA.

### Study design

This was a randomized controlled trial. Participants were randomized to participate in a TC LEE, 8-form TC or a stretching exercise for16 weeks. The outcome measures were BBS and TUG test scores, and COP measurements, assessed at baseline and at right after the intervention ended. The independent variable was different training (TC LEM, 8-form TC and stretching training) the participants received, and the dependent variables were all the measurements in this study.

### Protocol

**Fig. 2:**
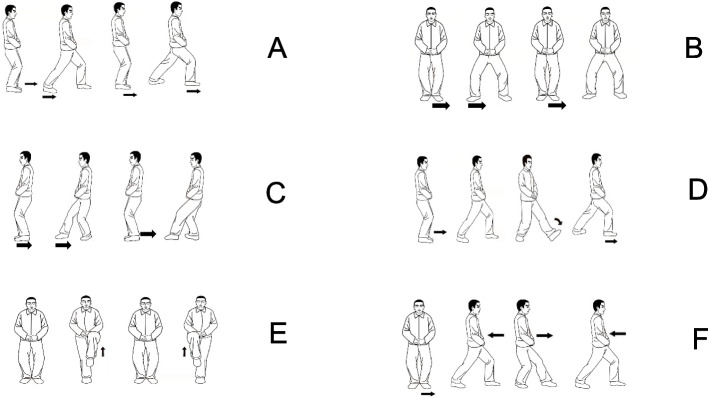
Tai Chi lower extremity exercise: Forward Stepping (**A**): the participant takes a step in the front left direction with the left foot. Once the left heel touches the ground, the participant moves the bodyweight from the right leg to the mid-point in between the feet. Then the participant repeats the movement on the right side. Side Stepping (**B**): the participant takes a step laterally to the left with the left leg. Once the left toes touch the ground, the participant shifts the bodyweight from the right leg to the left leg. Then the participant repeats the movement on the right side. Backward Stepping (**C**): the participant takes a step back with the left leg; once the left toe touches the ground, the participant moves the body weight backward from right leg to the left leg while lifting the right heel above the ground and pivoting the right forefoot to face forward. Then the participant repeats the movement on the right side. Turning Stepping (**D**): the participant takes a step in the front left direction with the left leg and shifts the bodyweight from the left leg to the mid-point between the feet. Then the participant takes a step in the front right direction with the right leg and shifts the bodyweight to the mid-point between the feet. Single-leg Stepping (**E**): the participant lifts the left leg up over knee height with the ankle slightly plantar flexed. While the left leg is being lifted, the participant fully extends the right knee. Then, the participant maintains this position for 3 s. Then the participant lowers the left leg down to the ground while flexing the right knee. The participant repeats the movement on the right side. Fixing Stepping (**F**): the participant takes a step to the left direction. Then the participant shifts the body weight between the 2 legs back and forth for 2 times. Then repeats the movement on the right side

TC LEE involved only the lower extremity movements of the 8-form TC [22], including: (A) Forward Stepping, (B) Backward Stepping, (C) Side Stepping, (D) Single-leg Stepping, (E) Turning Stepping, and (F) Fixing Stepping (Fig. 2). The difficulty of the TC LEE movements increased gradually, requiring progressively greater postural control and coordination. The participants were required to perform the TC LEE with their hands together in front of their waists throughout the entire exercise.

The 8-form TC was developed by Li and colleagues [16, 27], and consists of 8 poses selected from the 24-form Tai Chi. The 8 forms were named as: (1) Commencing Form, (2) Repulse Monkey, (3) Grasp Peacock’s Tail, (4) Wave Hands Like Clouds, (5) Fair Lady Works at Shuttles, (6) Golden Cock Stands on One Leg, (7) Brush Knees, and (8) Closing Form. Initial movements involved upper extremity movement patterns, such as Commencing Form, which required minimal postural control and whole-body coordination. The difficulty of the movements increased gradually, and required more postural control and coordination of the entire body, such as Brush Knees.

The stretching intervention included light walking, stretching, and relaxing. Each session began with 15 min of light walking (60% – 65% of the maximal heart rate) [28, 29] followed by 30 min of stretching of the major muscle groups of the entire body. After the stretching, participants completed 15 min of relaxation with deep breathing and meditation.

Ten experienced Tai Chi coaches led the TC LEE and the 8-form TC training. They all have more than 10 years of Tai Chi practicing, and they all have about 2 years of coaching experiences. Participants in the TC LEE group and the 8-form TC group had 4 training sessions per week for 16 weeks. Every training session lasted 1 h including 10 min of warm-up, 35 min of training, and 15 min of cool-down. First-aid certified research assistants assisted with coaching during each training session to ensure the safety of participants.

The participants in the stretching exercise group completed 4 training sessions per week for 16 weeks under the supervision of professional coaches. The participants were asked to maintain their usual lifestyles during this time period. Research assistants maintained weekly phone contact with all participants to ensure that they remained qualified for the study. Some participants reported they had regular light walking before training started, and the rest of the participants did not have regular exercise habits. Study participants from all 3 groups agreed to discontinue their light walking once they started the training sessions, and they had no additional exercise based on data from the daily exercise logs. Thus, all the participants had very limited weekly exercise before they started the training sessions, and they had no additional exercise while receiving the training in this study.

### Data collection

The outcome measures were BBS and TUG test scores, and COP measurements, which were collected before and immediately after the 16-week interventions.

#### BBS

A research assistant evaluated the participant’s performance on each test item for the BBS [30]. A score (0 – 4) was given for each item based on the quality of the performance and a total score was recorded after all items were completed.

#### TUG test

Participants completed the TUG test according to the instructions provided by the research assistants (Appendix 1) [31]. Three successful trials were collected for the TUG test.

#### COP measurements

For assessment of static postural control in quiet standing, the participant was instructed to stand upright with eyes open and feet together (no space between the feet) on a force plate (KISTLER, 9287BA and 9281CA, Switzerland) for 120 s. The participant was instructed to look straight ahead with arms beside the trunk in a comfortable position and stand as still as possible. Ground reaction force and moment data were collected at a sampling rate of 1000 Hz from which the COP variables were calculated. Each participant completed three successful trials in which the participant maintained the standing position as instructed without assistance and the data were collected appropriately. A 1-min break was provided between trials [32, 33]. Three successful trials were collected for data reduction. All research assistants in charge of data collection were blinded to the group assignments.

### Data reduction

The TUG test score was calculated by averaging the test score from 3 successful trials. The ground reaction force data were low-pass filtered at 12 Hz (4th order Butterworth) [34, 35]. The filtered data were used to calculate the root mean square (RMS) amplitude of COP displacement (mm) and the speed of the COP (mm/s) for each participant in anterior–posterior (AP) and medial–lateral (ML) directions (Appendix 2) [36]. The COP measurements were calculated by averaging the 3 successful trials for AP and for ML direction data.

### Data analysis

Intent-to-treat protocol was implemented in this study. Two-way ANOVAs (3 groups × 2 time points) with group as independent measure and time as repeated measure were performed to test the first hypothesis. If a significant group × time interaction effect was detected (*P*-value ≤ 0.05), 3 paired t-tests were conducted to compare the difference in each group between the 2 time points, and 2 independent t-tests were conducted to compare the difference between the stretching group and 2 intervention groups, respectively. The Bonferroni adjustment was used to control the Type I error. A *P*-value ≤ 0.01 was used to indicate statistical significance.

Change scores (the difference between before and after training) for the BBS and the TUG test were calculated for non-inferiority analyses. The 95% CI of the difference between the change scores from the 2 Tai Chi intervention groups was calculated, and the equivalence margin was determined as 60% of the minimal detectable change (MDC) for the BBS (3 × 0.6 = 1.8 points) and the TUG test (1.17 × 0.6 = 0.7 s) [37–39]. If the CI of difference between groups was within the equivalence margin, the TC LEE and 8-form TC groups were considered statistically similar.

If the dependent variables were significantly different at baseline among 3 groups, the change score (difference between pretests and posttests) for the dependent variable was calculated. One-way ANCOVAs with the baseline test as a covariate were then performed to test the hypotheses. Two independent t-tests (comparisons between intervention groups and stretching group, respectively) were conducted if a significant effect was found (*P* ≤ 0.05) for ANCOVAs. A *P*-value equal to or less than 0.025 was used to indicate statistical significance for follow-up tests.

A mixed design with time as a within-subject factor and groups as a between-subject factor was used to investigate the effects of intervention on the study variables. Based on previous literature, the effect size of Tai Chi on balance control of older adults with fall risk was medium to large effect size in the Berg Balance Scale and TUG test [11, 40, 41]. Assuming that differences in each dependent variable between pre- and post-intervention and among groups were no less than a medium effect size (0.35) in this study [11, 40–42], and the correlation coefficient among repeated measures was less than 0.50, the power analyses from Gpower software indicated 30 participants in each group with a probability of Type I error no less than 0.05 and a power of 0.8 [43].

$$N\approx 4\sum_{i=1}^{k}\sum_{j=1}^{k}{\rho }_{ij}{S}_{i}{S}_{ j} {\left({z}_{1-\frac{\alpha }{2}}+{z}_{1-\beta }\right)}^{2}/{(k\Delta )}^{2}$$

## Results

A total of 102 participants were enrolled and randomized into 3 groups (Fig. 1). Because the intent-to-treat protocol was implemented in this study, all participants were included in the analysis regardless of the dropout rate. No significant difference was found in the characteristics of the participants among the 3 groups (Table 1).

**Table 1:** Characteristics of participants at baseline

	**Group**			
**Variable**	TC LEE (*n* = 40)	8-form TC (*n* = 31)	Stretching (*n* = 31)	*P*-value
**Age (years)**	78.3 ± 7.4	79.9 ± 6.9	79.4 ± 3.6	0.596
**Height (cm)**	158.0 ± 8.0	160.0 ± 7.0	162.0 ± 9.0	0.119
**Weight (kg)**	60.9 ± 7.4	64.1 ± 8.4	62.2 ± 7.8	0.291
**Male/Female**	9/20	8/22	11/20	0.427
**MMSE Score**^a^	27.8 ± 1.9	28.4 ± 2.5	27.2 ± 2.8	0.541
**Mean Falls in Past Year**	2.0 ± 0.2	2.1 ± 0.3	2.0 ± 0.2	0.437
**Number of Slow Walking (%)**^b^	19 (65.5)	17 (56.3)	19 (61.3)	0.065
**Number of Finger Exercises (%)**^c^	10 (34.5)	14 (46.7)	12 (38.7)	0.058
**Number of Arthritis (%)**	6 (20.7)	9 (30.0)	7 (22.6)	0.677
**Number of High Blood Pressure (%)**	18 (62.1)	20 (66.7)	21 (67.7)	0.888
**Number of Hearing Impairments (%)**	2 (6.9)	1 (3.3)	2 (6.5)	0.807
**Number of Heart Diseases (%)**	15 (51.7)	20 (66.7)	21 (67.7)	0.365

^a^Mini-Mental State Examination score

^b^Slow walking was defined as a very light physical activity, during which the participants can breathe easily and carry on a conversation. Two times or less per week

^c^Finger exercise was a seated physical activity during which the participants exercise their hands and fingers. Two times per week

The overall attendance rate was 89.2 ± 8.4%. The mean attendance rate was 88.7 ± 8.6% in the TC LEE group, 88.8 ± 8.8% in the 8-form TC group, and 90.0 ± 7.9% in the stretching control group. Twelve participants dropped out during the training/follow-up tests, with 11 participants dropped out of the TC LEE group (*n* = 4, sick, *n* = 7, loss of interest), 1 participant dropped out of the 8-form TC group (*n* = 1, loss of interest) and no participant dropped out of the control group (drop rate was 27.5% for the TC LEE group, 3.2% for the 8-form TC group, and 0% for the control group).

No intervention safety issue was reported (falls, fractures, and joint pain etc. during or after training). Some participants expressed difficulty in learning the new movements in the first 2 training sessions in the 8-form TC group; however, these concerns resolved after the first 2 sessions as the participants became more familiar with the movements.

The BBS score was not significantly different among the 3 groups at baseline (*P* = 0.155). A significant interaction effect was identified for the BBS score (*P* = 0.002) among the 3 groups. The follow-up tests showed that at post-test, participants in the TC LEE group (*P* = 0.001) and 8-form TC group (*P* = 0.001) performed significantly better on the BBS than those in the stretching group (Table 2).

**Table 2:** BBS^1^ score, TUG^2^ test and COP^3^ comparisons among the 3 groups before and after training

	**BBS (point)**				**TUG Test (s)**				**RMS COP AP**^4^ **(mm)**				**RMS COP ML**^5^ **(mm)**				**COP Speed ML**^6^** (mm/s)**			
Time	TC LEE (*n* = 40)	8-form TC (*n* = 31)	Stretching (*n* = 31)	*P*-value	TC LEE (*n* = 40)	8-form TC (*n* = 31)	Stretching (*n* = 31)	*P*-value	TC LEE (*n* = 40)	8-form TC (*n* = 31)	Stretching (*n* = 31)	*P*-value	TC LEE (*n* = 40)	8-form TC (*n* = 31)	Stretching (*n* = 31)	*P*-value	TC LEE (*n* = 40)	8-form TC (*n* = 31)	Stretching (*n* = 31)	*P*-value
Before Training	50.6 ± 3.0	50.6 ± 2.3	49.7 ± 1.8	0.155	13.9 ± 2.5	13.3 ± 3.5	13.9 ± 1.6	0.657	6.1 ± 1.4	7.0 ± 1.8	6.7 ± 1.5	0.187	7.3 ± 2.3	8.0 ± 1.4	8.0 ± 1.0	0.396	20.2 ± 4.1	20.4 ± 6.5	21.5 ± 5.5	0.569
After Training	53.5 ± 2.1^a,b^	53.3 ± 1.0^a,b^	50.8 ± 1.7^b^	0.002	10.9 ± 2.4^a,b^	11.2 ± 2.0^a,b^	14.0 ± 2.3	0.001	3.9 ± 1.3^a,b^	4.3 ± 1.0^a,b^	6.6 ± 1.5	0.001	6.5 ± 1.5^a,b^	6.2 ± 1.2^a,b^	8.0 ± 1.1	0.001	13.1 ± 3.4^a,b^	12.3 ± 3.5^a,b^	21.5 ± 4.8	0.001
*P*-value	0.001	0.001	0.002		0.001	0.001	0.905		0.001	0.001	0.917		0.044	0.001	0.993		0.001	0.001	0.929	

*mm* : millimeter

*mm/s* : millimeter/s

^1^Berg Balance Scale

^2^Timed Up and Go test

^3^Center of Pressure

^4^Root mean square of center of pressure in anterior–posterior direction

^5^Root mean square of center of pressure in medial–lateral direction

^6^Center of pressure speed in medial–lateral direction

^a^The value is significantly different compared with the control group

^b^The value is significantly increased after the training

Non-inferiority analysis showed that the difference between the increases in the BBS scores of the TC LEE group and the 8-form TC group after training had a 95% CI between -0.8 and 1.3 points, which was within the equivalence margin of 1.8 points (Table 3). The changes in BBS score were equivalent between the 8-form TC group and the TC LEE group.

**Table 3:** Changes in BBS^a^ and TUG^b^ test in TC LEE group and 8-form TC group after training

	Change Score after Training		Difference of the Change Between Groups	95% CI of the Difference in the Change Between Groups
	TC LEE Group	8-form TC Group		
**BBS**	2.8 ± 2.1	2.6 ± 2.2	0.3 ± 0.5	-0.8 – 1.3^c^
**TUG test**	3.2 ± 2.0	2.2 ± 2.6	1.0 ± 0.6	0.1 – 2.1

^a^Berg Balance Scale

^b^Timed Up and Go test

^c^The upper limit of 95% CI of the difference was below the equivalence margin

The TUG test score was not significantly different among the 3 groups at baseline (*P* = 0.657). A significant interaction effect was identified for the TUG test score (*P* = 0.001). The follow-up tests showed that at post-test the TUG test scores of participants in the TC LEE group (*P* = 0.001) and the 8-form TC group (*P* = 0.001) were significantly decreased compared with those in the stretching group. The TUG test score of the stretching group did not significantly change after 16 weeks (*P* = 0.905) (Table 2).

Non-inferiority analysis showed that the difference between the changes in the TUG test score of the TC LEE group and the 8-form TC group after the training had a 95% CI between 0.1 and 2.1 s, which exceeded the equivalence margin of 0.7 s (Table 3). The changes in TUG test were not equivalent between 8-form TC group and TC LEE group, and greater decreases in the TUG test scores were observed in the TC LEE group.

The RMS amplitude of COP displacement in the AP direction was not significantly different among the 3 groups at baseline (*P* = 0.187). A significant interaction effect was identified for the RMS amplitude of COP displacement in the AP (*P* = 0.001) and ML (*P* = 0.001) directions. The follow-up tests showed that at post-test the TC LEE group and the 8-form TC group had significantly decreased RMS amplitude of COP displacement in the AP and the ML directions compared with the stretching group. The RMS amplitude of COP displacement in the AP direction (*P* = 0.917) and ML direction (*P* = 0.993) of the stretching group did not significantly change after 16 weeks (Table 2).

Average COP speed in the AP direction differed among the 3 groups at baseline (*P* = 0.025). One-way ANCOVA using the baseline value as a covariate detected a significant effect of group on the pre-post change scores for average COP speed in the AP direction (*P* = 0.001). Follow-up assessments indicated that the change scores for both the TC LEE and the 8-form TC groups were significantly greater than in the stretching group (Table 4).

**Table 4:** Comparisons of the changes of average COP AP speed^1^ among 3 groups before and after training (mm/s)

		**Group**		
Variable	TC LEE (*n* = 40)	8-form TC (*n* = 31)	Stretching (*n* = 31)	*P*-value
Change Score	7.4 ± 2.3^a^	7.1 ± 2.7^a^	3.9 ± 3.6	0.001

*mm/s:* millimeter/s

^1^Center of pressure speed in anterior–posterior direction

^a^The average COP AP (center of pressure in anterior–posterior direction) speed change is significantly different compared with the control group

A significant interaction effect was observed for the average COP speed in the ML direction (*P* = 0.001). The follow-up tests showed that at post-test the TC LEE group (*P* = 0.001) and the 8-form TC group (*P* = 0.001) had significantly slower average COP speed in the ML direction compared with the stretching group. The average speed of COP movement in the ML direction did not change in the stretching group (*P* = 0.929) (Table 2).

## Discussion

The purpose of this study was to compare the effects of two Tai Chi interventions (TC LEE and 8-form TC) and a stretching intervention on balance and functional mobility of older adults. The results fully support the hypothesis that both the TC LEE and the 8-form TC interventions would improve performance of the BBS, TUG, and COP measurements compared with a stretching control group. Furthermore, the results partially support the hypothesis that the TC LEE and 8-form TC interventions would result in similar improvements in BBS and TUG test performance.

The BBS and TUG test have been widely used to evaluate fall risk in older adults [44, 45]. The values obtained for the BBS and TUG assessments in this study were consistent with those reported in the literature [38, 46]. The improvement on the BBS and TUG tests indicate improvements in balance and mobility in older adults [47, 48]. Donoghue et al. reported a minimum detectable change of 3.3 points for the BBS at 95% confidence for participants who were in the 45 – 56 BBS score range [38]. The average BBS scores in TC LEE and 8-form TC groups increased by 2.7 ± 2.3 points and 2.6 ± 2.2 points, respectively, indicating that increases in the BBS in the TC LEE and 8-form TC group after intervention might be marginally meaningful. Similarly, Donoghue et.al reported that the minimum detectable change of the TUG test was 2.08 s [49]. The TUG test values in TC LEE and 8-form TC groups decreased by 3.2 ± 1.0 s and 2.1 ± 2.4 s, respectively, suggesting the improvements of TUG test after training in both Tai Chi groups were meaningful.

COP displacement and speed have been used in numerous studies as indicators of static postural control, and decreases in these outcomes indicate improved postural control [50–53]. Significant reductions in the RMS amplitude of COP displacement and the speed of COP were found in both Tai Chi intervention groups compared with the control group after training in this study, which was consistent with previous findings [54, 55]. Collectively, these findings suggest that both of the Tai Chi interventions evaluated in this investigation may improve balance and functional mobility in older adults.

Tai Chi movements have characteristics that might promote improved balance in older adults, such as slow movements, frequent body weight shifts in various directions, large range of motion of lower extremity joints, and maintenance of a semi-squat position, which require precise neuromuscular control for coordination [52]. Both the 8-form TC and the TC LEE forms include the beneficial characteristics mentioned above. These characteristics could potentially challenge the balance control of older adults, thereby improving the control of body weight over the base of support and improving the neuromuscular control of the lower extremities. The body weight shifts in the forward, backward, lateral, and diagonal directions mimic movements in daily life for older adults, thus potentially transferring to improved dynamic balance during movement. The combination of these improvements may, therefore, decrease falls risk of older adults.

Our results suggest that TC LEE produces similar improvements in the BBS and greater effects on the TUG test compared with the 8-form TC form. One unique characteristic of TC LEE is that it is performed without upper extremity movements, which potentially makes TC LEE movements more challenging to maintain balance using only the lower extremities. Upper extremity movements serve an important role in maintaining balance [56, 57]. Walking with restricted upper extremity swing is related to a less stable gait pattern, slower gait speed, and gait that is more susceptible to perturbations [56, 57]. Movements of the upper extremities can assist with balance corrections when perturbations occur [58]. Therefore, without movement of the upper extremities, balance might be more difficult to maintain. The Grasp Peacock’s Tail is an example of this. This form contains weight shifts between the legs back and forth. The upper extremities move in the same direction of shifts in bodyweight. These upper extremity movements might help practitioners move the trunk with changes in body weight movements and balance the momentum generated by the lower extremities. However, without assistance from the upper extremities, practitioners might need greater momentum from the lower extremities in moving the trunk towards the direction of shifts in body weight practicing the Fixing Stepping derived from the Grasp Peacock’s Tail. Thus, practitioners may need more precise neuromuscular control of their lower extremities to generate enough momentum for shifts in body weight, but not too much to cause a loss of balance. Consequently, the TC LEE might require more effort to maintain balance during training sessions. These findings might suggest that the lack of upper extremity movements in the TC LEE intervention might produce greater improvements in balance in older adults. Future studies investigating the role of upper extremity movements of Tai Chi should be done to test this hypothesis.

Falls usually occur in older adults when the upper extremities are occupied, such as carrying objects [59]. Older adults at heightened falls risk or with a history of falls also sway more when conducting secondary tasks with the upper extremities [60]. The TC LEE is practiced without upper extremity movements, which simulates the daily situations in which participants may have to maintain their balance without assistance from upper extremity movements.

The TC LEE could have practical and clinical benefits. TC LEE is practiced without arm movements, which may make Tai Chi movements easier to learn for older Tai Chi beginners, frail older adults, older adults with decreased cognitive capabilities, and older adults with upper extremity injuries who would like to improve their balance and prevent falls. TC LEE might require less cognitive resources because participants do not need to memorize the complicated combinations of upper extremity and lower extremity movements and the sequences of Tai Chi. Therefore, older adults can focus on lower extremity balance control when practicing this Tai Chi form. Practicing TC LEE could improve balance control and functional mobility, which can serve as a new option for clinicians to treat patients with increased risk of falls. Clinicians can teach patients the entire set of TC LEE movements, or they can select some of the stepping movements of interest for patients to practice. In addition, clinicians can combine TC LEE with existing fall prevention programs to provide effective, individualized, and multiple interventions for patients. Furthermore, TC LEE could be applied in different settings, such as, clinics, assisted living facilities, nursing homes and communities.

This study had some limitations that should be considered in the interpretation of the findings. The drop-out rate was greater in the TC LEE group compared with the other 2 groups. After interviewing all the participants who dropped out, many of the participants in the LEE group gave the reasons of drop-out was as follows. In this study, all the participants were deeply influenced by the Chinese culture. In their minds, Tai Chi involves fully body movements with upper and lower extremity movements. Participants in the TC LEE group may have had difficulty in connecting the TC LEE form with traditional Tai Chi. Since TC LEE was easy to learn, the participants spent about 3 weeks to learn and master it. Once they mastered it, they asked for full body Tai Chi training which could not be offered in the study. Thus, they gradually lost interest in participating in the study. The mean age of the participants in this study was around 80. Our subjects may not be representative of older adults in other age groups. Generalization of the findings of this study to the broader older adult population should be done with caution. This study also lacked data indicating the participants’ interest in practicing and difficulties encountered by participants in practicing the 8-form Tai Chi and TC LEE forms.

There are some strengths of this study. This study is a first step to determine the effective components of Tai Chi movements for older adults. This study could potentially simplify Tai Chi without sacrificing the fall prevention effects by introducing TC LEE. Randomized clinical trials with larger sample sizes should be done to explore further the effective components of Tai Chi in older adults with different ethnicity. Future studies could explore the other possible benefits of TC LEE on the balance control of older adults with different chronic conditions, such as stroke, Parkinson’s disease, and arthritis. Future studies should also investigate the interest and difficulties encountered by participants who engage in the simplified TC LEE form.

## Conclusion

In this study, we developed the TC LEE, which was performed with no upper extremity movements and with movement patterns that involved frequent shifts in body weight and frequent changes in stepping direction. TC LEE may simulate balance challenges encountered in daily activities by older adults. Based on the results of this study, TC LEE could be used as an effective balance training exercise for older adults.

### Supplementary Information

**Additional file 1.**

## Data Availability

The datasets generated and/or analysed during the current study were not publicly available due the participants’ requests to not share the data, but were available from the corresponding author on reasonable request.
