# Supplementary material for: The effect of Tai Chi lower extremity exercise on the balance control of older adults in assistant living communities
Source: BMC Complement Med Ther. 2024 Mar 6;24:112. doi: 10.1186/s12906-024-04382-9 (PMC10918873; doi:10.1186/s12906-024-04382-9)
Supplement: Supplementary file 1 — Additional file 1. [file 12906_2024_4382_MOESM1_ESM.docx]

**Appendix 1**

|  | Test | Score (0 – 4) |
| --- | --- | --- |
| 1 | Sitting to standing |  |
| 2 | Standing unsupported |  |
| 3 | Sitting unsupported |  |
| 4 | Standing to sitting |  |
| 5 | Transfers |  |
| 6 | Standing with eyes closed |  |
| 7 | Standing with feet together |  |
| 8 | Reaching forward with outstretched arm |  |
| 9 | Retrieving object from floor |  |
| 10 | Turning to look behind |  |
| 11 | Turning 360 degrees |  |
| 12 | Placing alternate foot on a stool |  |
| 13 | Standing with one foot in front |  |
| 14 | Standing on one foot |  |

**Table A1. Berg Balance Scale**

**“Timed Up and Go” test instruction**

The participant wears his or her regular footwear, and sits in a chair with armrests. A target line 3 meters from the front legs of the chair is drawn on the floor. The participant is instructed to stand up from the chair, walk to the target line at their normal pace, turn around at the target line, walk back to the chair at their normal pace, and sit down again. The rater starts timing the participant’s performance using a stopwatch on the word “Go” and stops timing when the participant sits down. The time is recorded to the nearest 0.1 second. An older adult who takes more than 12.0 seconds to complete the TUG test is considered at increased risk for falls. ^1^

**Appendix 2**

The root mean square (RMS) amplitude of COP displacement (mm) and the speed of the COP (mm/s) for each participant in AP and ML directions.

RMS amplitude of COP displacement (AP) = $\sqrt{\frac{\sum{(x_{i}-x)}^{2}}{N}}$ (1)

RMS amplitude of COP displacement (ML) = $\sqrt{\frac{\sum{(y_{i}-y)}^{2}}{N}}$ (2)

Average Speed of COP (AP) = $\frac{\sum_{i=2}^{N-2} \left| \frac{x_{i+1}-x_{i-1}}{t_{i+1}-t_{i-1}} \right|}{N-2}$ (3)

Average Speed of COP (ML) = $\frac{\sum_{i=2}^{N-2} \left| \frac{y_{i+1}-y_{i-1}}{t_{i+1}-t_{i-1}} \right|}{N-2}$ (4)

In the equations above, $x_{i}$ and $y_{i}$ were the position of COP at certain time point ($t_{i}$ ). $N$ was the number of data points. $x$ and $y$ were the average position of COP in AP and ML direction, respectively.

1. Schoene D, Wu SMS, Mikolaizak AS, Menant JC, Smith ST, Delbaere K, et al. Discriminative ability and predictive validity of the timed up and go test in identifying older people who fall: Systematic review and meta-analysis. *J Am Geriatr Soc.* 61. 2013:202-8.
